# Connexin Hemichannels in Astrocytes: Role in CNS Disorders

**DOI:** 10.3389/fnmol.2019.00023

**Published:** 2019-02-06

**Authors:** LingYan Xing, Tuo Yang, ShuSen Cui, Gang Chen

**Affiliations:** ^1^Key Laboratory of Neuroregeneration of Jiangsu and Ministry of Education, Co-innovation Center of Neuroregeneration, Nantong University, Nantong, China; ^2^Department of Hand Surgery, China-Japan Union Hospital of Jilin University, Changchun, China; ^3^Department of Anesthesiology, Affiliated Hospital of Nantong University, Nantong, China

**Keywords:** astrocyte, connexin 43, Alzheimer’s disease, glioma, ischemia, neuropathic pain

## Abstract

In the central nervous system (CNS), astrocytes form networks interconnected by gap junctions made from connexins of the subtypes Cx30 and Cx43. When unopposed by an adjoining hemichannel, astrocytic connexins can act as hemichannels to control the release of small molecules such as ATP and glutamate into the extracellular space. Accruing evidence indicates that astrocytic connexins are crucial for the coordination and maintenance of physiologic CNS activity. Here we provide an update on the role of astrocytic connexins in neurodegenerative disorders, glioma, and ischemia. In addition, we address the regulation of Cx43 in chronic pain.

## Introduction

As the most abundant cells in the central nervous system, astrocytes are critical for synaptic transmission and homeostasis maintenance. Astrocyte dysfunction has been associated with many neurological disorders, including but not limited to neurodegenerative diseases, gliomas, and ischemia (Verkhratsky et al., 2010; Yang et al., 2013; Hirayama and Koizumi, 2018). Over the last two decades, accumulating evidence has shown that astrocytes are also key mediators in pain development and maintenance (Ji et al., 2013).

Astrocytes in the CNS form a highly interconnected network via gap junctions or hemichannels. Each connexin hemichannel consists of six protein subunits termed connexins, which belong to a protein family encoded by 20–21 genes in mammals (Scott et al., 2012). An individual subunit possesses four alpha-helical transmembrane domains, connected by two extracellular loops and one intracellular loop, with cytoplasmic carboxyl and amine terminals (Bennett et al., 2016). When one hemichannel docks to its counterpart on the apposed cells, a gap junction is formed. Gap junctions of astrocytes allow rapid intercellular exchange of ions and metabolites, which is critical for K^+^ and glutamate buffering, calcium wave propagation, and synaptic plasticity (Li et al., 2014; Lapato and Tiwari-Woodruff, 2018). Unpaired connexins can act as hemichannels, which are responsible for the release of gliotransmitters, including ATP, glutamate, nicotinamide adenine dinucleotide (NAD), and D-serine to the extracellular milieu (Saez et al., 2003; Retamal et al., 2014). This offers a new exchange route between neurons and glia.

**FIGURE 1:**
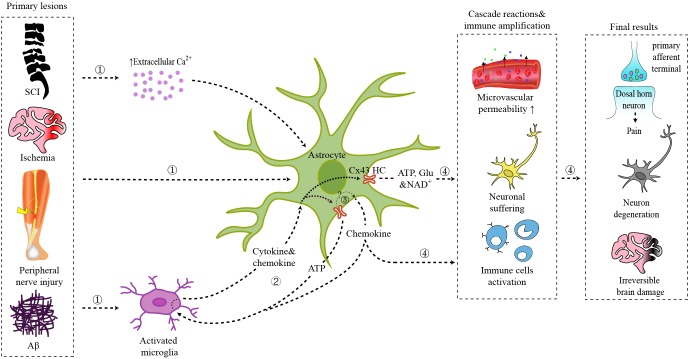
Schematic illustration of the role of astrocytic Cx43 in CNS disorders. 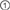 Primary lesions from spinal cord injury, brain ischemia, peripheral nerve injury, or Aβ plaques act on local astrocytes directly, triggering the interaction between activated microglia and astrocytes, or leading to an increase of extracellular Ca^2+^ concentration. 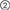 Interaction between microglia and astrocytes: ATP released by astrocytic Cx43 can act on microglia via P2X receptors; chemokines released by astrocytes can activate microglia; cytokines and chemokines released by activated microglia further trigger astrocyte activation and Cx43 HCs opening. 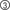 Cx43 HCs also mediate the synthesis and secretion of chemokines. 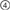 ATP, glutamate, NAD^+^, and chemokines released by Cx43 HCs modulate synaptic transmission directly or trigger the inflammatory cascade, which finally result in chronic pain, neuron degeneration, and irreversible brain damage.

Accruing evidence suggests that connexin hemichannels can open at both physiological and pathological conditions (Saez et al., 2003). The opening of hemichannels is highly dynamic and can be controlled by multiple regulators. Lower or higher intercellular Ca^2+^ can increase the opening probability of hemichannels (Decrock et al., 2011). In addition, pathological conditions, such as oxidative stress, lower pH, mechanical stimulation, and inflammation can significantly enhance the hemichannel opening (Johansen et al., 2011; Batra et al., 2014; Castellano and Eugenin, 2014; Retamal et al., 2015). The uncontrolled opening of hemichannels can lead to cell damage and homeostatic imbalance (Orellana, 2016). It is accepted that excessive release of ATP and glutamate or overload of intracellular free Ca^2+^ are toxic to neighboring cells or trigger secondary damages to distant cells (Takeuchi et al., 2006; Orellana et al., 2011). Of note, dysregulation of hemichannels permeability can also induce excessive influx of Na^+^ and Cl^-^, leading to osmotic and ionic imbalance (Orellana et al., 2016). In astrocytes, the predominant connexins are connexin 43 (Cx43), though Cx26 and Cx30 are also detectable (Rash et al., 2001a,b). This review will focus on the role of astrocytic Cx43 in the regulation of CNS disorders (Table 1, Figure 1). Furthermore, we also discuss an emerging role of astrocyte Cx43 in chronic pain (Table 2).

**Table 1:** Cx43 in the regulation of CNS disorders.

Diseases and models		Cx43 expression changes	Manipulations and drugs	Target channel	Mechanism	Outcomes	Reference
AD	APP/PS1/Gfap-Cx43 KO mice	↑	Astrocyte Cx43 KO	HC and GJC	APPswe/PS1dE9 + Gfap-Cx43 KO	↑cognitive function	Ren et al., 2018
						↓astrogliosis	
	APP/PS1/Gfap-Cx43 KO mice	↑	Astrocyte Cx43 KO	HC and GJC	APPswe/PS1dE9 + Gfap-Cx43 KO	↓gliotransmitter release	Yi et al., 2016
						↓neuronal damages	
	APPswe/PS1dE9 mice	↑	boldine	HC	HC blockade	↓hippocampal neuronal suffering	Yi et al., 2017a
	Astrocytes and acute hippocampal slices treated with the active fragment of Aβ(*in vitro*)	—	cannabinoids	HC	HC blockade	↓inflammatory profile evoked by Aβ	Gajardo-Gómez et al., 2017
Cerebral ischemia	Astrocyte from Wistar rats	Cx43 expression varies according to the time points and p-Cx43↑	carbenoxolone	GJC	GJC blockade	↑protective effects of ischemic preconditioning	Ma et al., 2018
	Neonatal SD rats	↑	Cx43 mimetic peptides Gap26 and Gap27	HC	Extracellular loop peptides	↓cerebral infarct volume	Li et al., 2015
Retinal ischemia- reperfusion	Wistar rats (*in vivo*) and endothelial cell (*in vitro*)	↑	Cx43 mimetic peptide5	HC	Extracellular loop peptides	↓dye leak (*in vivo*)	Danesh-Meyer et al., 2012
						↑retinal ganglion cell survival (*in vitro*)	
Glioma (glioblastoma)	Cx43 KO mice and Cx43 truncated mutant mice (Cx43K258stop)	↓as the glioma grade increases	Cx43 KO and Cx43 truncated	HC and GJC	Gfap:Cre+Cx43fl/fl; C-terminal truncation at amino acid 258	↓glioma invasion	Sin et al., 2016
	GBM cells (*in vitro*)	↑in the TMZ-resistant GBM cells	Cx43 siRNA	HC and GJC	RNA interference	↓TMZ resistance	Munoz et al., 2014
	BALB/c nude mice injected with LN229 human GBM stem cells	↑	αCT1	HC	Cx43 C-terminus mimetic peptide	↓TMZ resistance	Murphy et al., 2016
Myocardial ischemia	Intact heart of rats	—	Cx43 mimetic peptide Gap26	HC	Extracellular loop peptides	↑protection against myocardial ischemia–reperfusion injury	Hawat et al., 2010
Amyotrophic Lateral Sclerosis	SOD1 mice and SOD1^G93A^ mice	↑	Cx43 mimetic peptide Gap26	HC	Extracellular loop peptides	↑motor neurons survive	Almad et al., 2016


*↑denotes upregulation; ↓denotes downregulation; — denotes data unavailable.*

**Table 2:** Expression changes of Cx43 in different pain models.

Pain models and species	Cx43 expression changes	Manipulations and drugs	Target channel	Mechanism	Effects on pain	Reference
Spinal cord injury, mouse	↑	Cx43^-/-^Cx30^-/-^, double knockout	HC and GJC	Cx30-KO + Gfap:Cre Cx43^fl/fl^	↓heat hyperalgesia and mechanical allodynia	Chen et al., 2012
Spinal cord injury (thoracic spinal cord hemisection), mouse	↑	Intrathecal injection of fluorocitrate and carbenoxolone and Gap26	HC and GJC	astrocyte metabolic inhibitor;gap junction/hemichannel blocker; Cx43 extracellular loop peptides	↓SCI-induced bilateral below-level mechanical allodynia	Choi et al., 2016
Spinal cord contusion, rat	↑	Intraperitoneal injection of Peptide5	HC	Cx43 extracellular loop peptides	↓at-level mechanical allodynia	Mao et al., 2017b
Chronic constriction injury (CCI) of the sciatic nerves, mouse	↑	Intrathecal injection of CBX, Gap26 or Gap27, astroglial toxin pretreatment of astrocytes, or Cx43 siRNA	HC and GJC	GJC blockade; Cx43 extracellular loop peptides; RNA interference	↓mechanical allodynia	Chen et al., 2014
CCI, mouse	↑	—	—	—	—	Neumann et al., 2015
Spinal nerve ligation (SNL), rat	↓	Intrathecal Cx43 siRNA	HC and GJC	RNA interference	↓mechanical hypersensitivity	Xu et al., 2014
Spinal nerve ligation (SNL), rat	↑	Intrathecal CORM-2 administration	HC	Release CO as HC inhibitor	↓hyperalgesia and allodynia	Wang and Sun, 2017
Partial sciatic nerve ligation (PSNL), mouse	↓	Intrathecal injection of an adenovirus vector expressing Cx43	HC and GJC	RNA interference	↓PSNL-induced mechanical hypersensitivity	Morioka et al., 2015
PSNL, mouse	↓	Intrathecal injection of lycopene	HC and GJC	Reversed TNF-induced downregulation of Cx43 expression	↓mechanical hypersensitivity	Zhang et al., 2016
Inferior alveolar nerve injury, rat	↑	Administration of Gap27 in the trigeminal ganglion	HC	Cx43 extracellular loop peptides	↓mechanical hypersensitivity	Kaji et al., 2016
Chemotherapy (bortezomib -induced peripheral neuropathy), rat	↑	Intraperitoneal injection of minocycline and CBX	GJC	Glial activation inhibitor and gap junction decoupler	—	Robinson and Dougherty, 2015
Opioid intrathecal (i.t.) administration of morphine, rat	↑	Intrathecal injection of Gap26	HC	Cx43 extracellular loop peptides	↓morphine antinociceptive tolerance,	Shen et al., 2014
Inflammation (unilateral carrageenan (CA) injection), rat	↑	Intrathecal injection of CBX or Gap26	HC and GJC	Gap junction decoupler and Cx43 extracellular loop peptides	↓contralateral paw withdrawal frequency (PWF), while the ipsilateral PWF was not affected	Choi et al., 2017
Bone Cancer Walker 256 tumor cells inoculation into the tibia, rat	p-Cx43↑	Intrathecal injection of Gap26	HC	Cx43 extracellular loop peptides	↓mechanical allodynia	Hang et al., 2016
Bone Cancer (intra-femoral inoculation of Lewis lung carcinoma cells), mouse	↑	Intrathecal injection of CBX	GJC	Gap junction decoupler	↓pain hypersensitivity	Yang et al., 2018
Breakthrough cancer pain (BTcP), mouse	Cx43 protein↑ p-Cx43↓	Intrathecal injection of Gap26	HC	Cx43 extracellular loop peptides	↓pain hypersensitivity	Li et al., 2017


*↑denotes upregulation; ↓denotes downregulation; — denotes data unavailable.*

## Cx43 in Neurodegenerative Diseases

Alzheimer’s disease (AD), a representative CNS neurodegenerative disease characterized by plaques and tangles in the brain (Ballard et al., 2011), is the leading cause of dementia worldwide. Until recently, the role of astrocytes in AD has been appreciated, though astrocytic modification was discovered in AD decades ago (Nagy et al., 1996; Rodríguez et al., 2009; Verkhratsky et al., 2010). Astrogliosis and β-amyloid(Aβ) plaque, two prominent pathologic features of AD, are both highly associated with astrocytic connexins, which offer a novel pathological mechanism and a potential therapeutic target for AD (Yi et al., 2017b). Altered expression of astrocytic connexins have been observed in the brains of both AD patients and mice (Nagy et al., 1996; Mei et al., 2010), though the mechanisms by which connexins expression is changed, remain controversial. For example, the expression of Cx43 in astrocytic gap junctions of AD patients is upregulated in the cortical regions with Aβ plaques, and some plaques corresponded exactly to the potentiated Cx43 immunoreactive sites (Nagy et al., 1996). In older APP/PS1 mice, a murine model of familial AD, an increase of Cx43 and Cx30 immunoreactivity was found in 60–70% Aβ plaques of reactive astrocytes. However, a decrease in the expression of Cx43 and Cx30 was also found in a few newly formed plaques (<10%) (Mei et al., 2010). This discrepancy indicates that the alteration of Cx43 expression in AD depends on the amyloid pathology and local inflammatory status of the plaque sites (Koulakoff et al., 2012). The increase in Cx43 expression promotes astroglial activation and further alters astroglial channel function and the pathologic process of AD.

Studies have been performed to investigate the role of Cx43 in AD, either as gap junction channels or hemichannels. Interestingly, the reactive astrogliosis does not affect astroglial gap junctional communication in APP/PS1 mice (Yi et al., 2017b). Hemichannels in astrocytes, however, can be activated during the pathologic process of AD and are critical for the neuronal damage (Orellana et al., 2011). The activated hemichannels increase the release of ATP and glutamate from astrocytes around the amyloid plaques, leading to overload of neuronal Ca^2+^, synaptic depression (Pascual, 2005), and final neuronal damage (Yi et al., 2016). Neuronal damages in the process could be alleviated by Cx43 hemichannel blocker boldine or cannabinoids (Gajardo-Gómez et al., 2017; Yi et al., 2017a). In addition, in APP/PS1 mice, a specific deletion of astroglial Cx43 could significantly reduce astrogliosis and increase synapse numbers, though it had no effects on amyloid plaque formation or inflammatory response (Ren et al., 2018). These results indicate that Cx43 could be a novel therapeutic target for AD.

As the second most common chronic neurodegenerative disorder in the CNS, Parkinson’s disease (PD) possesses reactive astrocytes in the substantia nigra (Fernandez, 2012; Cabezas et al., 2014). Rotenone is a common neurotoxic substance used for generating PD experimental models. In both rotenone-treated rats and *in vitro* astrocytes, levels of both total Cx43 and phosphorylated Cx43 were elevated (Wang Y. et al., 2013). Additionally, gastrodin from a Chinese herbal medicine can ameliorate PD by downregulating Cx43 (Wang Y. et al., 2013). Cx43 expression was also found upregulated in patients with amyotrophic lateral sclerosis (ALS) or related models (Almad et al., 2016). This upregulated Cx43 expression led to elevated hemichannel activity, enhanced gap junction coupling and increased intracellular Ca^2+^ concentration, which contributed to motor neuron toxicity. Furthermore, it was conferred that Cx43 blocker or Cx43 hemichannel blocker provided protection against this neuron toxicity (Almad et al., 2016).

Overall, the role of astrocytes as well as astrocytic connexins has attracted more attention in the field of neurodegenerative diseases in recent years, due to their critical role in gliosis, inflammation, and neuronal damage (Freitas-Andrade and Naus, 2016). Here we pose several questions and perspectives for further study. First, in-depth studies may be needed to apply and clarify the targets of these interventions, for example, R76W mutant which specifically block gap junction channels (Xu et al., 2015), Gap19 (a specific Cx43 hemichannel blocker) (Wang N. et al., 2013) and non-selective peptides. Second, the discrepancy of Cx43 immunoreactivity in neurodegenerative diseases was found in a previous study (Mei et al., 2010), which may lead us to test how Cx43 works in a time-dependent manner.

## Cx43 in Glioma

Glioblastoma (GBM), a representative type of malignant glioma, is the most common and aggressive CNS malignant tumor (Sontheimer, 2015). Both the expression changes of Cx43 and its role in glioma progression are controversial, which may be attribute to high heterogeneity of this tumor (Sin et al., 2012). The expression of Cx43 varies with grades, stages, and locations of tumors. For example, Cx43 generally exhibits a lower expression in the tumor core within high-grade gliomas compared with low-grade ones (Sin et al., 2016). As a conventional therapeutic strategy, surgical resection supplemented with chemotherapy and radiotherapy confers a poor prognosis in patients with gliomas (Stupp et al., 2005). This poor prognosis is mainly caused by the resistance to the chemotherapeutic alkylating agents such as temozolomide (TMZ), and the invasive nature of the tumor cells (Sin et al., 2012, 2016; Wang et al., 2018). In the TMZ-resistant GBM cells, Cx43 expression showed a significant upregulation. Studies suggested that an increase of functional EGFR expression activated the JNK-ERK1/2-AP-1 axis to upregulate Cx43 expression in the TMZ-resistant GBM cells (Munoz et al., 2014). TMZ-resistance was significantly reduced when Cx43 was suppressed by peptides targeting Cx43 channels or Cx43 C-terminal (Gielen et al., 2013; Murphy et al., 2016; Grek et al., 2018), which implies TMZ-resistance is highly dependent on Cx43 in gliomas.

Temozolomide resistance may be mediated by Cx43 via the mitochondrial apoptosis pathway (Gielen et al., 2013), or interactions between Cx43 carboxyl terminus and actin cytoskeleton (Crespin et al., 2010). GBM cells treated with za restored TMZ sensitivity (Gielen et al., 2013; Murphy et al., 2016). These results indicated that Cx43 carboxyl terminus confers TMZ-resistance in gliomas. Cx43 carboxyl terminus promotes tumor cell migration, and therefore may contribute to glioma invasion (Bates et al., 2007). However, another study showed that Cx43 can promote tumor invasion via a carboxyl terminus-independent manner, since Cx43 without carboxyl terminus can also increase migration (Crespin et al., 2010), which may be derived from connexin-based Ca^2+^ signaling and ATP release (Sin et al., 2016). Notably, utilizing Cx43 peptidomimetics as an adjuvant for TMZ resistance has been proposed (Grek et al., 2018).

Not only does the expression of Cx43 change, but its role may also vary with grades, stages, and locations of the tumors. Since many studies focused on Cx43 in gliomas, many new therapeutic targets have been proposed (e.g., Cx43 extracellular loop, Cx43 loop/tail interactions, Cx43 C-terminal) (Delvaeye et al., 2018). The diverse effects of these drugs are needed to testify to different conditions. In addition, specific drugs targeting Cx43 for varying glioma, may be a better solution based on the dynamic changes of Cx43.

## Cx43 in Brain Ischemia

Brain ischemia is a leading cause of long-term disability or even mortality in adults. Insufficient blood flow, which fails to meet the high metabolic demands of the brain, will trigger the cascade reaction including tissue ischemia, reperfusion injury, inflammatory activity, leading to irreversible brain damage (Kim et al., 2018). Accumulating evidence suggests that astrocytic Cx43 expression is increased after hypoxia/ischemia injury and that Cx43 plays an important role in cell death and neuronal damage induced by cerebral ischemia (Davidson et al., 2012a,b, 2013, 2014; Ma et al., 2018). Ischemia/reperfusion injury and the following inflammatory activation can activate the astrocytic hemichannels via the increased extracellular Ca^2+^ and inflammatory factors released (Davidson et al., 2013). For instance, inflammatory factors just like IL-1β could reverse the inhibition of hemichannel activity caused by epidermal growth factor (EGF) (Morita et al., 2007); Cx43 hemichannels can also be triggered via p38 kinase by pro-inflammatory cytokines including IL-1β and TNF-α released by activated microglia (Retamal et al., 2007; Giaume et al., 2013). These abnormally opened hemichannels subsequently cause an uncontrolled release of ATP, glutamate, and an overload of Ca^2+^, leading to tissue excitotoxicity, amplification of the inflammation (Kim et al., 2018), and ultimately irreversible brain damage. The treatment of Cx43 mimetic peptide Gap26 leads to a reduction in both Cx43 expression and Cx43 hemichannels activity, which improve neurological function and reduce infarct volume (Li et al., 2015). According to this evidence, targeting to Cx43 might be a promising therapeutic strategy for brain ischemia, but more efforts are needed to develop specific inhibitors which can penetrate the blood-brain barrier.

## Astrocytes and Cx43 in Chronic Pain

Astrocytes are reactive in multiple types of conditioning-induced chronic pain, including peripheral and central nerve trauma, inflammation, chronic opioid exposure, etc. (Song and Zhao, 2001; Okada-Ogawa et al., 2009; Ren and Dubner, 2010; Chiang et al., 2012; Ji et al., 2013). Consistently, inhibiting the activity of astrocytes in the spinal cord can ameliorate chronic pain (Tsuda et al., 2011). Different from rapid activation of microglia, reactive astrocytes are usually found several days after injury and persist for a longer time (Mika et al., 2009; Old et al., 2015). This indicates that astrocytes might mainly contribute to the development and maintenance of chronic pain. The following mechanisms have been discussed in the astrocyte-mediated pain: (1) astrocytes can release multiple inflammatory mediators and neuromodulators, such as cytokines IL-1β, chemokines CCL2, and CXCL1; (2) a variety of receptors and transports, for example, ATP receptors P2XR and P2Y, glutamate transporter-1 (GLT-1), and glutamate and aspartic acid transporter (GLAST) can be activated in astrocytes; (3) the mitogen activated protein kinases (MAPKs) are also activated and further induce downstream signaling critical for pain; (4) reactive astrocytes also regulate the opening states of gap junctions or hemichannels, which further control the release of ATP, glutamate, and NAD^+^. These signaling pathways coordinate or interact with each other in response to pain. The role of Cx43 in pain is acknowledged by the study that Cx43/Cx30 deletion, instead of Cx30 single knockout, can alleviate the neuropathic pain developed between 4 and 8 weeks following spinal cord injury (Chen et al., 2012). Here, we will comprehensively discuss the role of Cx43 in chronic pain.

Studies have shown that Cx43 can act as a non-ligated hemichannel releasing small mediators or gliotransmitters, such as ATP and glutamate, into the extracellular environment (Bennett et al., 2003; D’hondt et al., 2014), which modulate synaptic transmission by directly interacting with nociceptive neurons, further contributing to pain. In addition, extracellular ATP also acts via its receptor P2X on non-neuronal cells, which contribute to pain by inducing the release of cytokines and chemokines.

Studies have shown that Cx43 can also regulate the expression or secretion of cytokines and chemokines in multiple systems. Although it is generally believed that hemichannels only allow passage of small molecules and ions, it has been reported that Cx43 can control the secretion of chemokine CXCL12 in bone marrow stromal cells (Schajnovitz et al., 2011). In the rat arthritis model, LPS treatment significantly enhanced Cx43 gene expression in rat fibroblast-like synoviocytes, whereas transfection of siCx43 inhibited the LPS-induced overexpression of pro-inflammatory cytokines and chemokines (Tsuchida et al., 2013). Furthermore, CBX (carbenoxolone, a non-selective gap-junction inhibitor) reduces the increase of IL-1β and IL6 in cerebrospinal fluid caused by intrathecal injection of HIV1 gp120 (Spataro et al., 2004). The expression and secretion of cytokines or chemokines are up-regulated in TNF-α-activated astrocytes, which are important in the induction and maintenance of pain hypersensitivity (Gao et al., 2009; Huh et al., 2017). Our previous study has shown that TNF-α-induced CXCL1 and CCL2 release from astrocytes, were blocked by Cx43 small interfering RNA, CBX and ^43^Gap26 or ^37,43^Gap27 (two Cx43 mimetic peptides that blocks hemichannels), indicating that astrocytic Cx43 hemichannels are responsible for the release of the chemokines (Chen et al., 2014). Another study showed that intrathecal injection of ^43^Gap26 markedly attenuated mechanical allodynia in rat bone cancer model and reduced CXCL12 production from spinal dorsal horn in astrocytes (Hang et al., 2016). Although this evidence suggests that Cx43 hemichannels mediate the synthesis and secretion of chemokines, the mechanism remains unclear as chemokines are too large to directly efflux through the Cx43 hemichannels. One possible explanation is that activated calcium signaling contributes to CXCL12 secretion via the GTPase RalA (Schajnovitz et al., 2011). Another possibility is that an increase of purine induced by Cx43 may regulate the release of chemokines, based on the role of purinergic signaling in astrocytic release (Chen et al., 2014). Moreover, the Cx43 hemichannel may be hyperactive in pathological conditions, thereby causing chemokines to “leak” out of astrocytes through cytoskeletal changes (Cotrina et al., 2000).

The expression changes of Cx43 following pain is still inconclusive (Table 2). Multiple studies have shown that Cx43 is upregulated in astrocytes following nerve ligation and spinal cord injury, and that inhibition of Cx43 can attenuate pain hypersensitivity (Wu et al., 2011; Chen et al., 2012, 2014, 2018; Shen et al., 2014; Neumann et al., 2015; Robinson and Dougherty, 2015; Choi et al., 2016; Hang et al., 2016; Kaji et al., 2016; Mao et al., 2017a,b; Wang and Sun, 2017; Yang et al., 2018). On the contrary, few studies showed that a decrease of Cx43 following nerve injury could contribute to pain hypersensitivity (Xu et al., 2014; Morioka et al., 2015; Zhang et al., 2016). Interestingly, in a mouse model of breakthrough cancer pain, Cx43 protein level is upregulated while phosphorylation of Cx43 (p-Cx43) is downregulated (Li et al., 2017). The conflict might come from distinct pain conditions or models. Additionally, it might not be the Cx43 expression level alone that determines the enhanced or attenuated function of hemichannels or gap junctions, because even in the pain model with decreased CX43 expression, inhibition of CX43 function can still relieve pain (Xu et al., 2014).

Studies have shown that Cx43 can be highly regulated by astrocytic inflammatory mediators, growth factors, or receptors. TNF-α and IL-1β, inflammatory mediators produced by astrocytes in pain, can modulate the expression of Cx43 (Morioka et al., 2015; Choi et al., 2017). For example, TNF decreases Cx43 expression in naive mice, which can be reversed by the TNF inhibitor (Morioka et al., 2015). Reactive astrocytes also lead to an increase in the basic fibroblast growth factor (bFGF or FGF-2) in the late phase following nerve injury. bFGF increases the expression of Cx43 and enhances intercellular communication of Cx43 gap junction in cardiac fibroblasts (Doble and Kardami, 1995), though the study of the role of bFGF in astrocytic Cx43 is lacking. In addition, the sigma-1 receptor activated by astrocytes in both peripheral and central neuropathy, could also modulate the activation of Cx43. The increase in Cx43 expression can be reversed by a sigma-1 receptor blocker (Choi et al., 2016).

Increasing evidence has shown that mitogen-activated protein kinases (MAPKs) can also regulate the opening states of Cx43 channels in astrocytes. Activation of MAPKs family members contribute to pain sensitization. For instance, extracellular signal-regulated kinases (ERKs) is significantly upregulated in astrocytes when animals were injected with complete Freund’s adjuvant (CFA), a drug inducing inflammation and pain (Weyerbacher et al., 2010). Phosphorylation of C-Jun N-terminal kinases (JNKs), predominantly JNK-1, is observed in spinal astrocytes in a persistent pain condition (Gao et al., 2010). MAPKs lead to a closure of CX43 gap junction and hemichannels, while MAPK phosphatase make Cx43 preferentially open (Kim et al., 1999; Goodenough and Paul, 2003; Solan and Lampe, 2005). Notably, phosphorylation and dephosphorylation events not only regulate the gating of channels, but also the trafficking and assembly of connexins (Ribeiro-Rodrigues et al., 2017), indicating the complicated effects of MAPKs on Cx43.

Though the interaction between Cx43 and molecules involved in pain offers a complicated feedback loop in pain development and maintenance, based on the current studies, strategies to suppress the function of Cx43 may be a robust approach for pain relief (Wu et al., 2011; Chen et al., 2012, 2014; Shen et al., 2014; Xu et al., 2014; Neumann et al., 2015; Robinson and Dougherty, 2015; Choi et al., 2016; Hang et al., 2016; Kaji et al., 2016; Mao et al., 2017a,b; Wang and Sun, 2017; Yang et al., 2018).

Cx43 might function as hemichannels or a gap junction, which has not been clearly characterized in every single study (Tables 1, 2). A non-selective gap-junction inhibitor carbenoxolone (CBX) can reduces neuropathic pain (Wang et al., 2014), supporting the ideas that Cx43 can function as a gap junction. On the other hand, studies proposed a model in which Cx43 can function as a non-junctional hemichannel to release mediators such as ATP and glutamate (Bennett et al., 2003; D’hondt et al., 2014). There are a few ways to distinguish hemichannels and gap junctions. For example, when used for short incubation time or at a low concentration, peptide 5, a mimetic of Cx43, can only inhibit hemichannels, but when applied for a long incubation time or at a long concentration, can attenuate both hemichannels and gap junctions (Choi et al., 2016; Mao et al., 2017a). Another approach to distinguish Cx43 gap junction and hemichannels are using the dyes uptaken. Lucifer yellow, only permeable to the gap junction, while ethidium bromide is considered exclusively uptaken by hemichannels. The diffusion rates of these dyes will differentiate hemichannel and gap junction. Interestingly, a peptide derived from the cytoplasmic loop of Cx43 termed Gap19 can specifically function in Cx43 hemichannels while not affecting gap junctions (Abudara et al., 2014). In addition, La3+ can specifically block hemichannels rather than gap junctions. Typically, the use of these drugs is accompanied by other blockers or dyes, however, thus far no drugs that only target to Cx43 are available.

The enriched expression of Cx43 and wide distribution of astrocytes in the brain and spinal cord might explain the complicated phenotypes observed in animals with Cx43 manipulation. Though changes of either ATP, glutamate, or Ca^2+^ can be detected when Cx43 is manipulated, it is hard to tell how each molecule contributes to the system since (1) few studies have systematically tested all changes of these molecules; (2) not only local signaling is affected by these small molecules.

## Conclusion and Perspectives

Astrocytes modulate extrasynaptic or synaptic milieu to further enhance or dampen electrochemical signaling propagating in neurons. As the major connexin altered following nerve injury, Cx43 can be regulated by multiple signals under pathological conditions, which in turn, modulate several downstream signals critical for neuronal activity, and further contribute to a variety of CNS disorders, including pain. So far, up to 21 phosphorylation sites have been reported in Cx43 (Pogoda et al., 2016), indicating a complex post-translational modification. Further studies might be necessary to characterize how these phosphorylation sites contribute to specific CNS disorders. Of note, besides connexin hemichannels, pannexin is also detectable in astrocytes (Garre et al., 2010). Pannexin-1, like Cx43, can also be inhibited by the non-selective gap-junction blocker CBX (Garre et al., 2010). Therefore, the role of Pannexin-1 in pain control could be an interesting topic to explore. In addition, how connexins interact with pannexins and the signaling pathways mediated by connexins or pannexins need further investigation. Selective manipulation of connexins might be a potential therapeutic approach in some CNS disorders.

## Author Contributions

LX and TY wrote and drafted the manuscript. All authors contributed to manuscript revision, read and approved the submitted version.

## Conflict of Interest Statement

The authors declare that the research was conducted in the absence of any commercial or financial relationships that could be construed as a potential conflict of interest.
